# The Pfam protein families database: embracing AI/ML

**DOI:** 10.1093/nar/gkae997

**Published:** 2024-11-14

**Authors:** Typhaine Paysan-Lafosse, Antonina Andreeva, Matthias Blum, Sara Rocio Chuguransky, Tiago Grego, Beatriz Lazaro Pinto, Gustavo A Salazar, Maxwell L Bileschi, Felipe Llinares-López, Laetitia Meng-Papaxanthos, Lucy J Colwell, Nick V Grishin, R Dustin Schaeffer, Damiano Clementel, Silvio C E Tosatto, Erik Sonnhammer, Valerie Wood, Alex Bateman

**Affiliations:** European Molecular Biology Laboratory, European Bioinformatics Institute (EMBL-EBI), Wellcome Genome Campus, Hinxton CB10 1SD, UK; European Molecular Biology Laboratory, European Bioinformatics Institute (EMBL-EBI), Wellcome Genome Campus, Hinxton CB10 1SD, UK; European Molecular Biology Laboratory, European Bioinformatics Institute (EMBL-EBI), Wellcome Genome Campus, Hinxton CB10 1SD, UK; European Molecular Biology Laboratory, European Bioinformatics Institute (EMBL-EBI), Wellcome Genome Campus, Hinxton CB10 1SD, UK; European Molecular Biology Laboratory, European Bioinformatics Institute (EMBL-EBI), Wellcome Genome Campus, Hinxton CB10 1SD, UK; European Molecular Biology Laboratory, European Bioinformatics Institute (EMBL-EBI), Wellcome Genome Campus, Hinxton CB10 1SD, UK; European Molecular Biology Laboratory, European Bioinformatics Institute (EMBL-EBI), Wellcome Genome Campus, Hinxton CB10 1SD, UK; Google DeepMind, 355 Main Street, Cambridge, MA 02142, USA; Google DeepMind, Brandschenkestr. 110, Zurich 8002, Switzerland; Google DeepMind, Brandschenkestr. 110, Zurich 8002, Switzerland; Google DeepMind, 355 Main Street, Cambridge, MA 02142, USA; Department of Chemistry, University of Cambridge, Lansfield road, Cambridge CB2 1EW, UK; Department of Biophysics, University of Texas Southwestern Medical Center, 5323 Harry Hines Blvd., Dallas, TX75390, USA; Department of Biochemistry, University of Texas Southwestern Medical Center, 5323 Harry Hines Blvd., Dallas, TX75390, USA; Department of Biophysics, University of Texas Southwestern Medical Center, 5323 Harry Hines Blvd., Dallas, TX75390, USA; Department of Biomedical Sciences, University of Padova, Via 8 Febbraio, 2, 35122 Padova, Italy; Department of Biomedical Sciences, University of Padova, Via 8 Febbraio, 2, 35122 Padova, Italy; Institute of Biomembranes, Bioenergetics and Molecular Biotechnologies, National Research Council (CNR-IBIOM), Via Giovanni Amendola, 122/O, 70126 Bari, Italy; Department of Biochemistry and Biophysics, Stockholm University, Science for Life Laboratory, Tomtebodavägen 23A, 17165 Solna, Sweden; Department of Biochemistry, University of Cambridge, Hopkins Building Downing Site, Tennis Court Road, Cambridge CB2 1QW, UK; European Molecular Biology Laboratory, European Bioinformatics Institute (EMBL-EBI), Wellcome Genome Campus, Hinxton CB10 1SD, UK

## Abstract

The Pfam protein families database is a comprehensive collection of protein domains and families used for genome annotation and protein structure and function analysis (https://www.ebi.ac.uk/interpro/). This update describes major developments in Pfam since 2020, including decommissioning the Pfam website and integration with InterPro, harmonization with the ECOD structural classification, and expanded curation of metagenomic, microprotein and repeat-containing families. We highlight how AlphaFold structure predictions are being leveraged to refine domain boundaries and identify new domains. New families discovered through large-scale sequence similarity analysis of AlphaFold models are described. We also detail the development of Pfam-N, which uses deep learning to expand family coverage, achieving an 8.8% increase in UniProtKB coverage compared to standard Pfam. We discuss plans for more frequent Pfam releases integrated with InterPro and the potential for artificial intelligence to further assist curation. Despite recent advances, many protein families remain to be classified, and Pfam continues working toward comprehensive coverage of the protein universe.

## Graphical Abstract

**Graphical Abstract:**
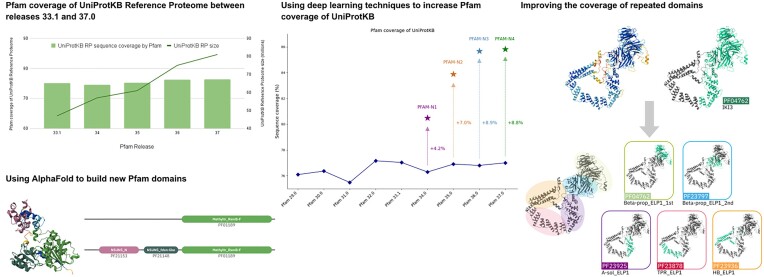

## Introduction

Pfam is a comprehensive database of protein families and domains that is widely used for the analysis of novel genomes and metagenomes. It serves as a crucial tool for guiding experimental research on specific proteins and systems. Each family entry within the Pfam database contains a seed alignment containing a representative set of sequences, from which a profile hidden Markov model (HMM) is generated. This HMM is then used to search against a sequence database called *pfamseq* (containing sequences from the UniProtKB reference proteomes [RPs]) using the HMMER software (http://hmmer.org/) (1). Sequence regions that meet a family-specific curated threshold, known as the gathering threshold, are aligned to the profile HMM to create a full alignment. Pfam entries are annotated by our curators with functional information sourced from the literature, where available.

Although the underlying sequence database used to build Pfam alignments is based on the UniProtKB RP, all the profile HMMs are also searched against the whole of UniProtKB, and the resulting matches are made available on the InterPro website (https://www.ebi.ac.uk/interpro) and in flat file format on the Pfam ftp (https://ftp.ebi.ac.uk/pub/databases/Pfam/releases).

When a Pfam entry is created or updated, we search it against *pfamseq*, trying to identify more distant homologs. Pfam entries are designed to avoid overlaps, ensuring that the same region of a sequence does not match more than one family. This non-overlap rule is an important quality control measure, helping to prevent the inclusion of false positive matches within a family. However, since Pfam 28.0, we relaxed this rule to allow small overlaps between families, as resolving all overlaps during each *Pfamseq* update has become increasingly time-consuming (2). There are many situations, such as incorrect gene predictions due to skipped exons or fusions of adjacent genes that cause overlapping of models, that are extremely difficult to resolve.

Sets of Pfam entries that are believed to be evolutionarily related are grouped into clans (3). Relationships between entries are identified through sequence similarity, structural similarity, functional similarity, and/or profile-profile comparisons using software such as HHsearch (4). Where possible, a single, comprehensive profile HMM is built to detect all members of a family. For some of the larger superfamilies, where this is not feasible, multiple-profile HMMs are built and placed within the same clan. Since families within a clan are evolutionarily related, they are allowed to overlap with other members of the same clan.

The development of advanced structure prediction methods has profoundly impacted the construction of Pfam entries. High-accuracy structure predictions, particularly those generated by tools such as AlphaFold (5), have significantly enhanced our ability to identify and characterize protein families. These predictions provide crucial insights into the 3D conformations of proteins, which in turn inform the delineation of evolutionary relationships and functional annotations. By incorporating structural data, we can more accurately define domain boundaries, detect distant homologs and resolve ambiguities that arise from sequence-based analyses alone.

Here, we describe the work undertaken since Pfam release 33.1, including the decommissioning of the Pfam website, our collaboration with the ECOD database (6), the utilization of structure predictions to construct new Pfam entries and enhance existing ones, and some of the family updates implemented in Pfam release 37.0. We also detail the development of Pfam-N, which uses deep learning to expand the coverage of Pfam families.

### Decommission of the Pfam website

In 2022, we took the decision to retire the Pfam website. The Pfam website codebase was first released over 20 years ago, and although it has been updated from time to time, some of its core functionality still dates to its origins. The Perl codebase represented a significant technical debt, and it was becoming progressively harder to maintain. Additionally, the most time-consuming part of the Pfam release pipeline was the generation of data exclusively related to the website rather than the core data of Pfam, i.e. its alignments and models. To address this issue, we decided to use the InterPro website as the primary way to view Pfam data. This would mean that European Molecular Biology Laboratory, European Bioinformatics Institute (EMBL-EBI), hosted a single website for protein family-related information rather than two. The InterPro website (https://www.ebi.ac.uk/interpro) has been redesigned using up-to-date technologies, including a modern framework (React) (7), and Pfam data and different viewing features are now available on the InterPro website.

**Figure 1.**
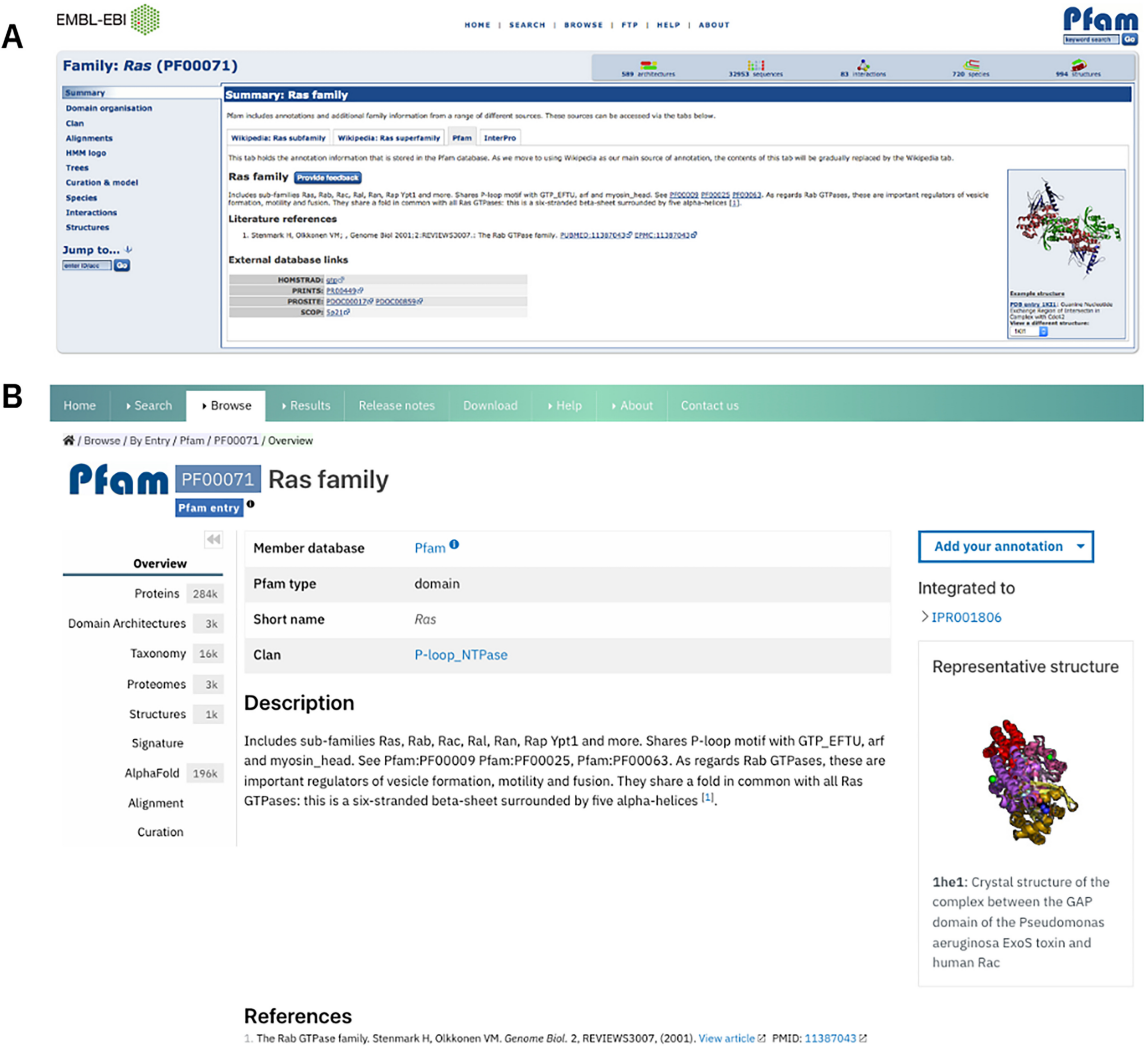
Example of a Pfam family page for the Ras domain (Pfam: PF00071) on the Pfam legacy website (**A**) and on the InterPro website (**B**).

The decommission was carried out over the course of a year. The first step consisted of expanding the InterPro website to incorporate several features from the Pfam website that were lacking, including a compact protein domain view with a set of non-overlapping representative domains for each protein. This work is detailed in the paper describing InterPro in this issue (Blum *et al.*, 2025). The second phase consisted of announcing the decommission to encourage Pfam users to start their transition to the InterPro website. Multiple announcements were made on the Pfam website, Pfam blog and social media platforms. We also ran a webinar highlighting where to find Pfam data on the InterPro website (Figure 1) to allow a smooth transition of Pfam users toward using the InterPro website.

With the decommission of the Pfam website, we have also slightly changed the MySQL database schema, as some tables were only used by the Pfam website. The change has been made from Pfam release 36.0 onward.

Although we tried to carefully explain our approach to users, there has been a persistent misunderstanding that because the Pfam website was being shut down, the Pfam database itself was being terminated. As Mark Twain said after erroneous press reports of his death, ‘The report of my death was an exaggeration’. We hope that this paper will help to quell the exaggerated rumors of our demise.

### Update of the Pfam documentation and online training materials

With the decommissioning of the Pfam website, we have undertaken an extensive update of the Pfam documentation (https://pfam-docs.readthedocs.io/en/latest/index.html) to assist users in locating relevant information on the InterPro website. Additionally, we have revised the Pfam online training materials (https://pfam-docs.readthedocs.io/en/latest/training.html), providing an important resource for users to become familiar with the concepts of Pfam and to find relevant information.

### From Pfam release 33.1 to release 37.0

Since 2020 and the release of Pfam version 33.1, we have had four new releases of Pfam. Despite the growth of the UniProtKB RP, which has almost doubled in size, Pfam has been able not only to maintain its sequence coverage but has even increased it by 0.8% since Pfam release 33.1 (Table 1). We also see that the residue coverage has decreased by 0.5%, presumably due to an increase in the number of eukaryotic proteomes available that overall have a lower residue coverage.

**Table 1.** Summary of Pfam release content and UniProtKB Reference Proteome (RP) coverage

Pfam Release	Total number of families	Total number of clans	Number of new families	Number of new clans	UniProtKB RP size (number of sequences)	UniProtKB RP sequence coverage by Pfam	UniProtKB RP residue coverage by Pfam
33.1	18 259	635	355	8	47 million	75.1%	49.3%
34.0	19 179	645	935	11	57 million	74.5%	48.8%
35.0	19 632	657	460	12	61 million	75.2%	48.7%
36.0	20 795	660	1191	5	75 million	76.2%	48.6%
37.0	21 979	709	1196	50	81 million	76.3%	48.8%

In the following sections, we describe some of the efforts to increase the content of Pfam in several important areas.

### Metagenomic protein families

To increase the Pfam coverage of metagenomic sequence space, we have created 710 metagenomic protein families. These families were built by co-clustering the MGnify (8) and UniProtKB (9) protein sequences and generating candidate profile-HMMs for potential inclusion in Pfam. This pipeline used MMseqs2 (10) to carry out the clustering of MGnify and UniProtKB, which generated a set of 434 651 340 clusters. We kept clusters with at least 1 UniProtKB and 1 MGnify sequence for which no Pfam had previously been assigned and automatically generated 10,000 candidate Pfam families that were put forward for curation. In general, our approach is to select the largest families after searching the HMMs against UniProtKB RP. We found there are still many large families in metagenomics data that are poorly represented in UniProt. As UniProt’s representation of genomics data improves, we will likely revisit building further families with this strategy.

### Harmonizing the Pfam and ECOD classifications

With the production of AlphaFold models for essentially the entirety of UniProtKB (5), the goals and scope of both sequence and structure classification have essentially converged. Whereas historically, databases like ECOD (Evolutionary Classification of Protein Domains) and CATH (Class (C), Architecture (A), Topology (T), and Homologous Superfamily (H)) have focused only on structures from the PDB, they are now beginning to classify proteins where only structure predictions are available. Given this background, we saw an opportunity to collaborate more closely. We work with the ECOD team to harmonize the Pfam and ECOD classifications. We developed a method to identify inconsistencies between the Pfam and ECOD classifications, detect missing Pfam families, identify incorrect domain boundaries in Pfam, which led to splitting or merging existing Pfam entries, and assign clan membership to existing Pfams. In Pfam 36.0, we built 638 new Pfam entries to cover PDB entries which didn’t have a Pfam annotation but had an ECOD annotation. Based on this work, the ECOD database replaced its ECODf collection of HMMs with Pfam HMMs. This work is described in the paper describing ECOD in this issue (Schaeffer *et al.*, 2025). Creating new Pfam families based on the ECOD classification means we can easily identify relationships between existing families and the new ones, grouping them together into clans. This greatly facilitates the addition of families to existing clans or the creation of new ones. For example, we created the THUMP clan based on ECOD (THioUridine synthases, RNA Methylases and Pseudouridine synthases CL0747), which in Pfam 36.0 included three families: the existing THUMP domain (Pfam: PF02926), the THUMP domain of eukaryotic Pus10 (such as human Pus10 Q3MIT2 included in Pfam: PF21237) and the Ribosomal RNA large subunit methyltransferase M, THUMP-like domain (Pfam: PF21239). The THUMP domain is involved in RNA metabolism and is present in enzymes involved in at least three unrelated types of RNA-modification.

### AlphaFold-driven curation

**Figure 2.**
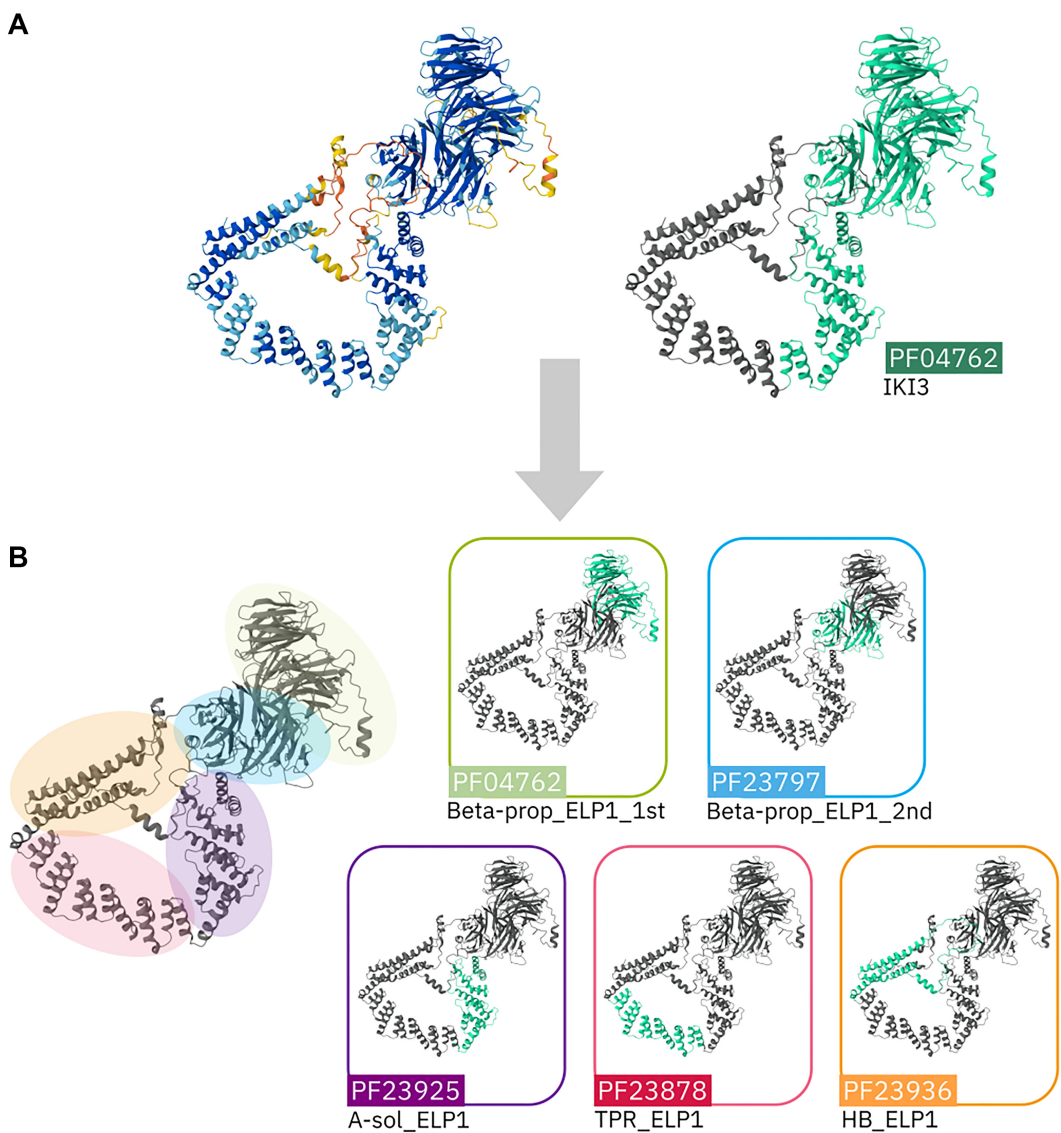
Refinement of boundaries for Pfam entry PF04762 using AlphaFold prediction for human ELP1 (UniProtKB: O95163). (**A**) The AlphaFold model of ELP1 colored by pLDDT score (left) and by the original incorrect domain boundaries of Pfam entry PF04762. (**B**) Representation of the new boundaries for PF04762 and new Pfam entries for the additional domains of this protein (PF23797, PF32925, PF23878, and PF23936).

AlphaFold has transformed the fields of protein structure and classification. Since its initial release, we have used AlphaFold-predicted structures to determine functions for previously uncharacterized domains, identify missing domains and refine the boundaries of Pfam domains. An example of AlphaFold-guided refinement of boundaries of Pfam entries is PF04762, which includes the Elongator complex protein 1 (ELP1, UniProtKB: O95163) from humans and its homologs. This Pfam model covered 70% of ELP1’s length and, from the AlphaFold prediction for this sequence, it was possible to split the model and create new entries to represent each of the domains in this group of proteins, which consist of two β-propellers at the N-terminus followed by a long, helical C-terminal dimerization domain. In Figure 2A, the AlphaFold prediction for ELP1 is displayed with the old match of PF04762 highlighted in green. Figure 2B shows the representation of the domains after the refinement of boundaries, including the newly created ones, which will be available in the next Pfam release. The annotations for the C-terminal dimerization domains of ELP1 (PF23925, PF23878 and PF23936) were also supported by (11).

**Figure 3.**
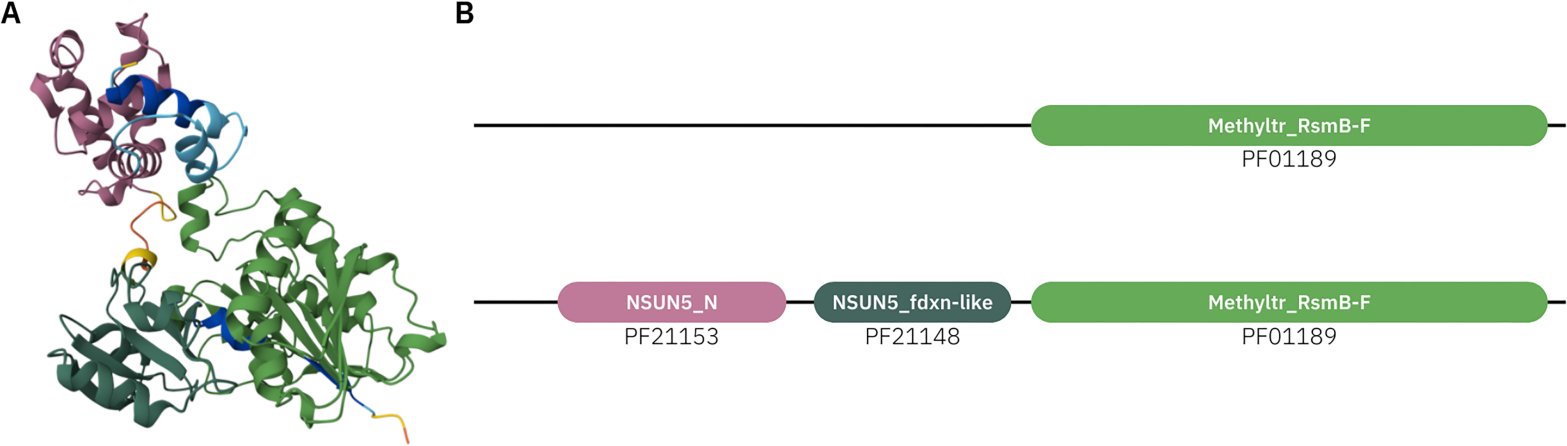
The NSUN5 protein (UniProtKB: Q96P11) with enhanced Pfam domain coverage. (**A**) AlphaFold structure prediction with the three domains highlighted: N-terminal, middle, and C-terminal. (**B**) Pfam annotations before (top) and after (bottom) the AlphaFold structure prediction. Two additional domains have been annotated: N-terminal (Pfam: PF21153) and middle (Pfam: PF21148) domains.

In collaboration with ECOD using their Domain Parser for AlphaFold Models (DPAMs) (12), we have been able to generate missing Pfam domains and refine the boundaries of existing domains. As a result, 114 new Pfam families were created up to Pfam release 37.0. For example, in the case of the NSUN5 28S rRNA (cytosine-C(5))-methyltransferase (UniProtKB: Q96P11), the methyltransferase domain existed (Pfam: PF01189), but the N-terminal (Pfam: PF21153) and middle (Pfam: PF21148) domains were missing Pfam annotations (Figure 3).

### New families and folds in the natural protein universe

We collaborated with members of the University of Basel/SIB who carried out a large-scale sequence similarity analysis of UniProt representatives with high-confidence AlphaFold models. The aim was to identify, prioritize and annotate novel protein families, superfamilies and folds (13). A key aspect of this work was the creation of a sequence similarity network, which allowed us to examine clusters of functionally ‘dark’ proteins and discover 146 new Pfam families. A significant fraction (133) of these newly identified families are domains of unknown function. Nonetheless, within these ‘dark’ families, we identified many intriguing cases, some of which are detailed below.

### DUF6919 (PF21897)

**Figure 4.**
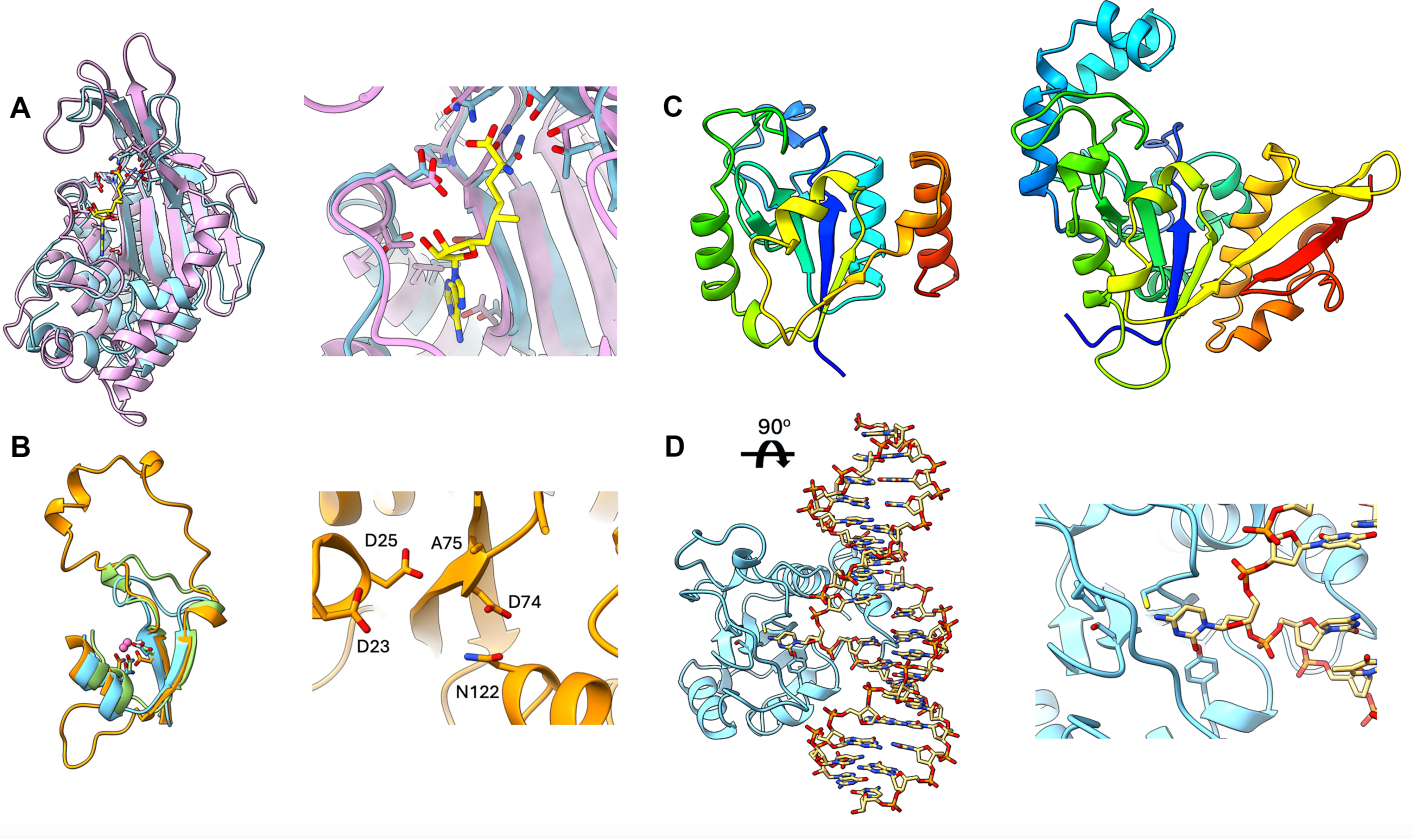
(**A**) Left: Superposition of the crystal structure of the C-terminal SAM-binding regulatory domain of human MTHFR reductase bound to S-adenosylhomocysteine (SAH) (PDB: 6fcx, pink) and the AlphaFold2 model of an uncharacterized protein from *Streptomyces candidus* (UniProtKB: A0A7 × 0HLX8, light blue), a member of DUF6919. Right: Close view of the SAM-binding site of MTHFR. Residues conserved in eukaryotic MTHFR and in the DUF6919 SEED alignment are shown in sticks. SAH is shown in yellow. (**B**) Left: Superposition of the HNH minimal β-β-α structural core of the AlphaFold model of uncharacterized protein from *Arthrobacter sp*. (UniProtKB: A0A1S9MJR2, orange) and the crystal structures of the rare-cutting HNH restriction endonuclease PacI (PDB: 3m7k, light blue) and the CRISPR-associated Cas9 endonuclease from *Acidothermus cellulolyticus* (PDB: 8d2k, green). Metal ions are shown in pink. Right: Close view of the A0A1S9MJR2 putative active site. The invariant putative metal coordinating (D74 and N122) and catalytic (D23 and D25) residues are shown in sticks. (**C**) A side-by-side comparison of the left: the AlphaFold2 model of uncharacterized protein YfjM from *Bacillus subtilis* (UniProtKB: O31547), a member of DUF6884 and the right: the crystal structure of DNA-binding protein YaaA from *E. coli* (PDB: 5caj). Both structures are rendered in cartoon format and colored in a rainbow gradient (blue to red, indicating N- to C-terminal end), highlighting structure similarities. (**D**) Left: AlphaFold3 model of YfjM bound to a DNA-RNA hybrid (view from the top after a ∼90° rotation around the *x*-axis compared to the structure shown in (C), left). Right: Close view of the putative YfjM active site with a cytosine, flipped and positioned near its conserved constituent residues. The latter are shown in sticks. The figures were rendered using ChimeraX (16).

One interesting finding was DUF6919, which is found as a standalone domain in bacteria. Its function is unknown, but it has a detectable similarity to the C-terminal S-adenosylmethionine (SAM)-binding regulatory domain of 5,10-methylenetetrahydrofolate reductase (MTHFR, Pfam: PF21895). The regulatory domain of MTHFR follows the catalytic domain, and this two-domain architecture is typical only for eukaryotes. In MTHFR, this domain binds SAM, but its fold is topologically distinct from the classical SAM-dependent methyltransferases and non-methyltransferases (14,15). Members of DUF6919 are predicted to share these unique topological features, comprising two antiparallel side-by-side-arranged β-sheets elaborated with α-helices. Besides the common structural core, DUF6919 and MTHFR are likely to have commonality in function, given the strict conservation of residues within the DUF6919 family that are also found in equivalent positions to residues constituting the eukaryotic MTHFR SAM-binding site (Figure 4A).

### DUF6994 (PF22507)

Another example discovered during the collaboration is DUF6994 (Pfam: PF22507), which is predicted to share common structural features with type IV restriction endonucleases and members of the HNH domain superfamily, demonstrating another interesting evolutionary finding. The HNH protein domains are found in Cas9, BrxU and related proteins, which are part of bacterial defense systems. The active sites of these enzymes are built around a minimal β-β-α fold that coordinates a catalytic metal ion. A typical feature is the so-called ‘omega loop’ (17), connecting the β-strands, which could vary in size but still retain a very distinct ‘omega-like’ conformation. This unique minimal β-β-α fold was identified in DUF6994 (Figure 4B, left). The conservation of the putative active site residues in DUF6994 is not strict, particularly the position of the catalytic histidine, which is replaced by alanine. The presence of two invariant aspartate residues that point toward the putative active site suggests that these proteins may still function as enzymes but probably via a somewhat different catalytic mechanism (Figure 4B, right). They may still retain their catalytic activity by employing these conserved aspartates, which in the predicted AlphaFold2 models are well positioned to undertake a role in a potential nucleophilic attack.

### DUF6884 (PF21818)

DUF6884, found either as a standalone protein or in combination with other domains, contains two highly conserved CxxxK and LSAxxG sequence motifs that may constitute a part of an active site. DUF6884 is distantly related to the YaaA protein from *Escherichia coli* (Pfam: PF03883), a DNA-binding protein that is a key player in the oxidative stress response (18). Besides a common structural core comprising a distinct α/β ‘cantaloupe’ fold, DUF6884 and YaaA share a common positively charged cleft that is probably involved in DNA-binding and recognition (Figure 4C and Supplementary Figure S1). In addition, a member of DUF6884, YfjM from *Bacillus subtilis*, has been recently identified among several interactors of DNA damage lesions (abasic and RNA base), suggesting that this protein could be involved in DNA repair (19). This hypothesis is supported by a high-confidence AlphaFold3 prediction (ipTM 0.83), in which YfjM recognizes the base-pair mismatch and flips a nucleotide into its putative active site (Figure 4D). The model of the predicted YfjM and DNA-RNA hybrid complex is available in ModelArchive at https://www.modelarchive.org/doi/10.5452/ma-ddli5.

### Annotation of microproteins

A microprotein is a small protein comprising 100 amino acids or fewer, encoded by a small open reading frame (smORF). Microproteins have diverse functions but are not well represented in Pfam for several reasons: first, they are frequently overlooked by gene prediction methods; second, their short length poses challenges in identifying homologs due to low signal-to-noise ratios; and third, their experimental isolation and characterization can be challenging. To address this underrepresentation, two strategies were employed. First, microproteins were identified within the UniProtKB Swiss-Prot dataset. Second, the EuropePMC API was queried using a range of filters to locate relevant information in the scientific literature. As a result, we identified 119 families of microproteins not previously represented in Pfam. Notable examples include microproteins involved in the regulation of larger proteins, such as Mitoregulin, which enhances fatty acid β-oxidation (Pfam: PF22002) (20), and the MgtS protein, which increases intracellular magnesium levels (Pfam: PF22865) (21,22).

### Update on Pfam-B

In addition to our HMM-based Pfam entries (Pfam-A), we provide a set of unannotated, computationally generated multiple sequence alignments called Pfam-B. It was created by the same pipeline that has been used since release 33.1, which uses MMSeqs2 (10) run with the cluster option and bidirectional coverage mode. It contains domain families with at least 20 member sequences, for which multiple sequence alignments were generated with FAMSA (23). This resulted in 177 011 Pfam-B families that, on average, contain 125 sequences (maximum 35 574) and are 432 amino acids in length (maximum 27 820). As previously, Pfam-B is only released as alignments in a tar archive on the Pfam FTP site (file Pfam-B.tgz). The pipeline prunes and prioritizes the families such that the first entries are the most conserved and largest alignments, which have the highest chance of representing useful novel domain families that may be turned into Pfam-A entries.

### Increasing the coverage of repeats in Pfam

The evolutionary history of proteins has been dominated by the processes of duplication and divergence of sequence. When these duplications occur within a protein, it leads to the phenomenon of protein repeats. Repeats are extremely common in proteins, both at the level of sequence and even more so at the level of protein structure. Understanding the repeat structure of a protein can help better understand its function, structure and evolution. Many of the largest Pfam clans, such as the β-propeller (CL0186) and TPR (Tetratricopeptide-like repeats, CL0020), contain repeats, showing the evolutionary success of repetitive regions. However, repeat families also present significant challenges to create, meaning that the coverage of repetitive proteins in Pfam is not as good as it could be. This was addressed through two collaborative projects detailed below.

### Collaboration with RepeatsDB

RepeatsDB is a database providing information about tandem repeats in protein structures (24) and provides an excellent resource to better understand the coverage and quality of Pfam repeat annotation. RepeatsDB provides detailed annotation of the span of individual repeats within known structures. In the earliest days of Pfam, we tried to build HMM models that could detect individual repeats of short repeats, such as TPRs and β-propeller blades. Some of these models still exist. However, we have progressively moved toward having models that represent multiple repeats because these models are simply more sensitive and able to retrieve many more homologs. However, this is at the expense of the ability to identify individual repeats. The comparison of RepeatsDB and Pfam allows for cross-validation, improving annotations with orthogonal information to increase the robustness of our annotation.

When comparing Pfam and RepeatsDB, ideally, both annotations should match, i.e., a given type of structural repeat should have its own HMM, and a given Pfam domain known to be repeated should produce its own structural repeat. Despite the effort produced by both resources, this is still not true. Cross-validation of Pfam domains and repeat regions in RepeatsDB starts with the definition of coverage. Coverage describes the fraction of overlapping residues between the repeat annotations of Pfam and RepeatsDB on a given protein sequence, with either database providing the reference.

If the ideal setting applies, we would expect the coverage of Pfam domains in RepeatsDB regions to be very high, possibly reaching 100%. Regions with low coverage are worth investigating, as they might mean that a Pfam or RepeatsDB annotation is not correct or that there is some missing data that needs to be included. Either case can potentially expand the information held in both databases.

**Figure 5.**
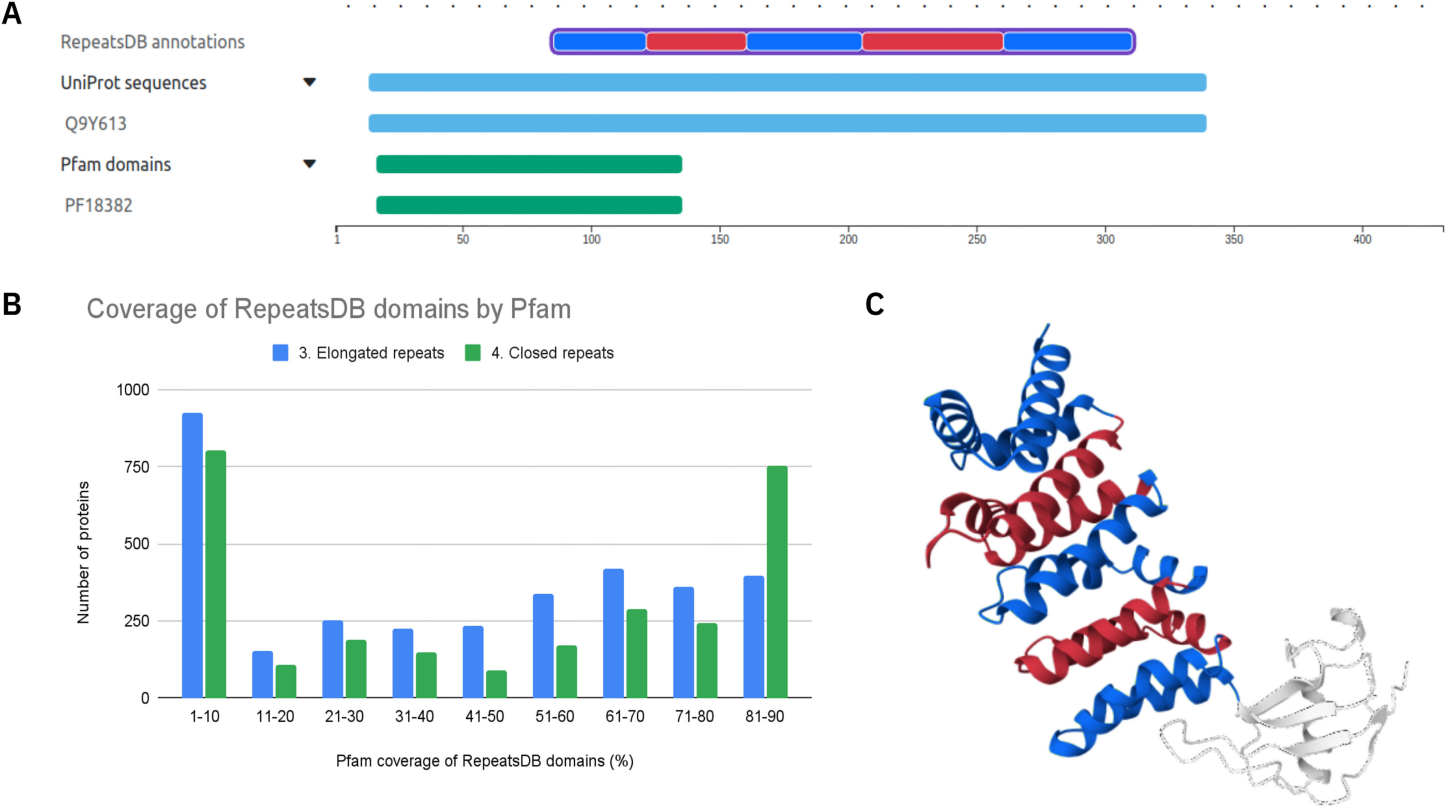
Coverage of Pfam domains on RepeatsDB regions. (**A**) Sequence annotation overview for human FH1/FH2 domain-containing protein 1 overlaid with the RepeatsDB (first line) and Pfam (third line) annotations. Pfam only annotates the N-terminal region, roughly corresponding to the first repeat unit. (**B**) Histogram showing the coverage of the protein sequence by Pfam in bins of 10% for elongated (class 3) and closed repeats (class 4) in RepeatsDB. (**C**) The structure of human FH1/FH2 domain-containing protein 1 (UniProtKB: Q9Y613) is shown with alternating coloring of the RepeatsDB repeat units.

The results of our analysis are shown in Figure 5B. The horizontal axis represents the coverage of any Pfam domain on each manually curated region in RepeatsDB in bins of 10%. RepeatsDB class 3 (elongated repeats) is shown in blue, and class 4 (closed repeats) is shown in green. Elongated Repeats are made of 5 to 40 residues where multiple tandem repeat units are required for the stable structure of the protein, while closed repeats are made of 30–60 residues where a relatively fixed number of repeat units are needed to form a stable domain structure. These repeats are generally arranged in a circular manner; they are the most representative classes in RepeatsDB (24). The vertical axis represents the number of proteins found in each bin. The distribution for closed repeats is skewed for high Pfam coverage, indicating that the latter captures them well. However, for both classes, there are a large number of proteins where the overlap is 10% or less, indicating that some RepeatsDB regions are poorly covered by Pfam and are worth investigating. Among the regions poorly covered by Pfam, the repeated units of PDB 3dad chain B are correctly identified in RepeatsDB, as shown in Figure 5C. However, the region is poorly covered by Pfam PF18382, identified as matching on the same protein structure through SIFTS (25), which covers only 9% of the repeated region, as shown in Figure 5A. To resolve these discrepancies, PF18382 should be truncated at the C-terminus to end before the repeated region, and a new Pfam entry should be created to cover the repeated region.

Our analysis also identified large Pfam domains, which have a large coverage of multiple different entries in RepeatsDB. In these cases, the Pfam entry contains multiple domains, indicating that it should be split into multiple entries. This type of adjustment was made in Pfam 37.0. for the WD40 domain on PDB 5nzu chain A, mapped to UniProtKB entry Q8CIE6 through SIFTS.

These examples prove the utility of a cross-validation process involving Pfam and RepeatsDB databases to analyze proteins from two different perspectives. The process is applicable to future releases of either Pfam or RepeatsDB, allowing increased coordination of the data.

### REFRACT project

Pfam was a partner in the REFRACT (Repeat protein Function, Refinement, Annotation and Classification of Topologies) project. This was a collaborative initiative between European and Latin American institutions, funded by the Marie Skłodowska-Curie Horizon 2020 program of the European Union. The project aimed to improve the understanding of tandem repeat proteins and establish a standardized classification and best practices for these proteins.

As part of this international consortium, EMBL-EBI hosted ten interns between 2019 and 2024; six of them contributed significantly to the curation of Pfam, focusing on improving and expanding the annotations of repeat-containing proteins. During this period, a total of 185 new Pfam entries were created, 73 of which were classified as repeats, with β-propellers and TPRs being the most common (29 and 20 entries, respectively). Most of these new entries will be included in the upcoming Pfam release.

**Figure 6.**
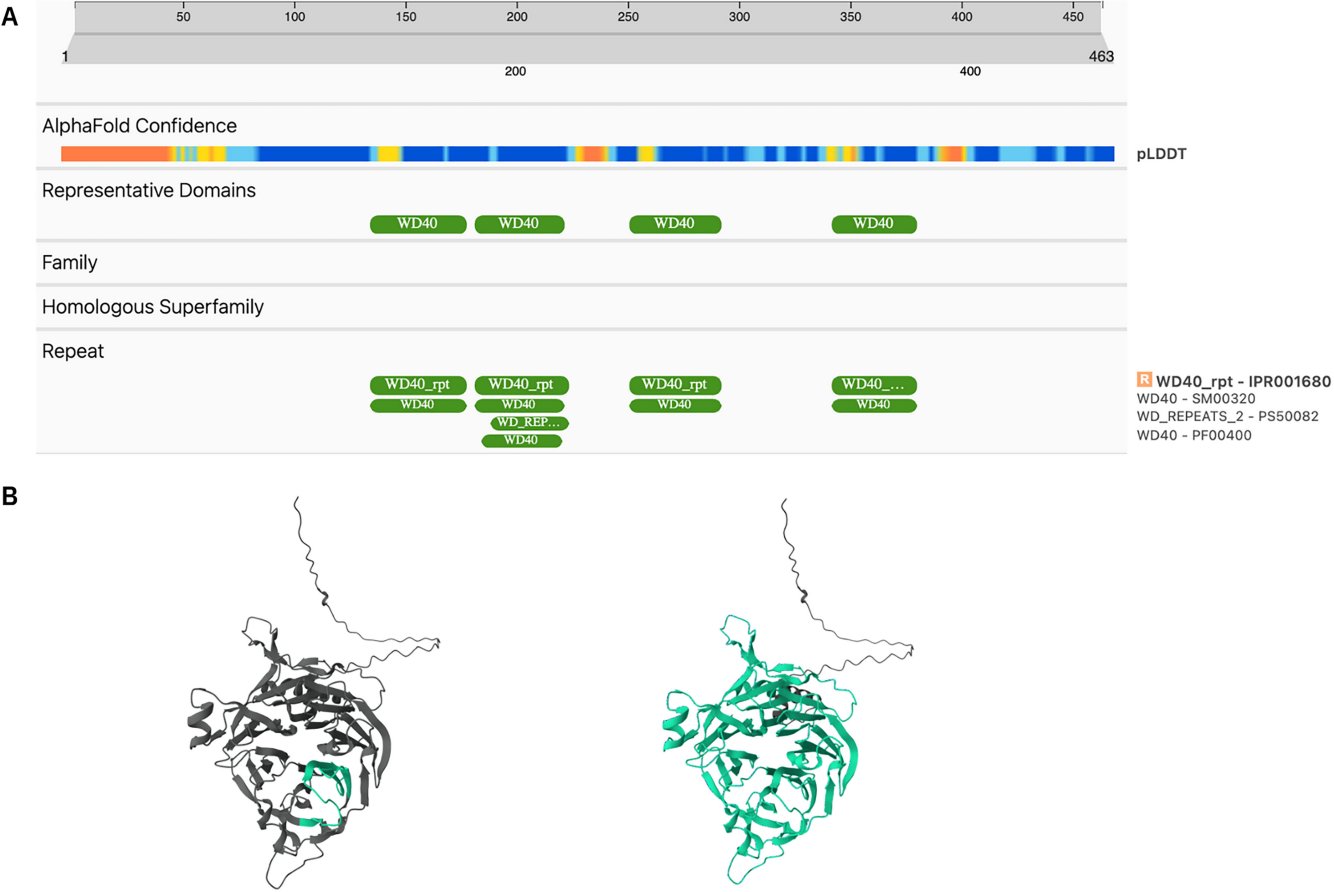
(**A**) Previous annotations for Q5VW00 in InterPro, where PF00400 covered only one repeat. (**B**) Pfam coverage for Q5VW00, PF00400 on the left, and new Pfam coverage for PF23760 on the right.

The primary focus of the interns was the refinement of beta-propeller annotations. By adopting the approach that builds functional domains consisting of multiple repeats instead of individual repeat units, Pfam models are more consistent and accurate, reducing false positives and providing more functional annotations for related proteins. For example, the DDB1- and CUL4-associated factor 12-like protein 2 from humans (UniProtKB: Q5VW00) was initially represented by only one repeat in Pfam PF00400. Given that PF00400 is a large entry, encompassing over 750 000 sequences (including 2000 Swiss-Prot sequences), improving its annotation presented a significant challenge. To address this, new Pfam entries were created to encapsulate the entire β-propeller domain WD40 (Figure 6), allowing the annotation of more closely related proteins and offering more specific and useful information for the scientific community.

### Using Deep Learning to expand Pfam (Pfam-N)

In 2019, Dr Lucy Colwell’s team released a preprint detailing a novel deep learning approach that was trained on Pfam data and improved upon it, which enhanced the performance of the HMMER software. Since 2020, we have collaborated with Dr Lucy Colwell’s research team at Google DeepMind to expand the Pfam coverage through deep learning techniques. This research has resulted in the creation of Pfam-N (N denoting network).

### Pfam-N version 1

An ensemble model called ProtENN (ENN for Ensemble of Neural Networks) was developed. The model was trained on a set of sequences with known Pfam protein family hits. From what was learned by the convolutional neural network, the model was able to predict Pfam protein families hits for sequence regions not found in the training set. However, the method required being given a domain-sized region of a sequence because the method could only predict a single Pfam label per sequence. Additional Pfam protein families hits were identified by looking at sub-threshold hits from the HMMER package against UniProt sequences and scoring them against ProtENN. After applying the model to the statistically insignificant hits of Pfam HMMs, Pfam-N was released for the hits that ProtENN confidently assigned a domain to (26). This first iteration of Pfam using deep learning led to an increased coverage of the UniProtKB RP 2020_06 by 4.2% compared to Pfam 34.0.

### Pfam-N version 2

In 2022, following the release of Pfam 35.0, ProtENN was retrained, leading to an increased coverage of 8.5% of the UniProtKB RP compared to Pfam 35.0 coverage (https://tinyurl.com/protenn2). The model’s training methodology was adapted from the Pfam-N version 1 approach, with a key modification in the prediction method. Instead of using HMMs to identify a ginel Pfam family hit for a sequence, the network employed a residue-level classification strategy. This approach assigned probabilities to each individual amino acid residue, indicating its likelihood of belonging to any of the 19 632 Pfam families or 655 clans in the database. When a contiguous length of 20 residues or more is predicted to belong to the same family, a domain call for that family is ‘called’ by the neural network. We found that many false positives from the network were removed by suppressing predictions from overlapping non-homologous families (i.e. families in different clans).

### Pfam-N version 3

**Figure 7.**
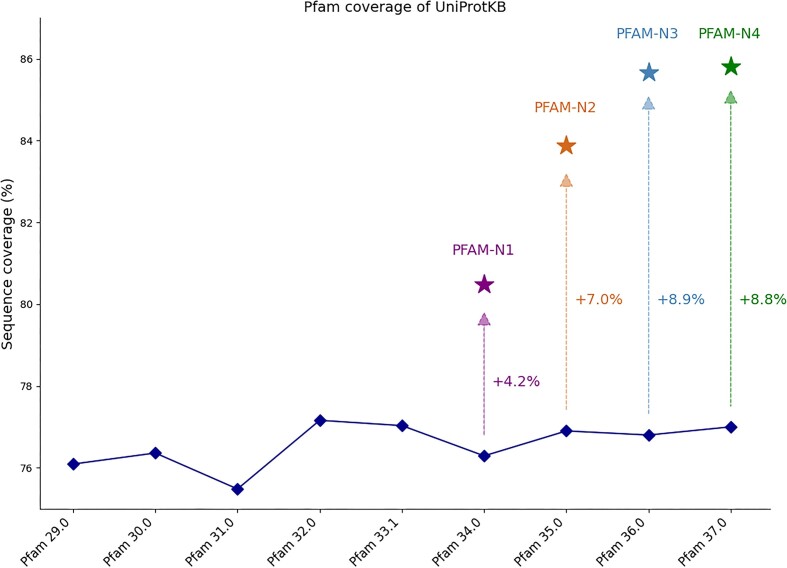
Comparison of the sequence coverage of UniProtKB between Pfam, Pfam-N versions 1 and 2 (Convolutional neural network) and Pfam versions 3 and 4 (Maskformer).

In 2023, the Google Research team developed an end-to-end transformer-based segmentation model inspired by the computer vision literature, showing an important increase in performance, significantly enhancing the accuracy of the predictions and coverage. The new model, called InterPro-N, is trained on the 13 member databases of InterPro, which enables the model to learn the relationships between them, improving consistency. The model is inspired by Maskformer (27), widely used in computer vision for segmentation tasks. The Pfam-N data that have been released corresponds to the Pfam predictions only. The new method, initially trained on InterPro 96.0, including Pfam 36.0, has achieved a coverage of 85.7% of UniProtKB 2024_03, a gain of 8.9% compared to Pfam 36.0. Following the release of InterPro 100.0 and Pfam 37.0, the model was retrained and has achieved a coverage of 85.8% of UniProtKB 2024_04, a gain of 8.8% compared to Pfam 37.0. In comparison, the previous version of Pfam-N showed a coverage increase of 7% compared to Pfam 35.0 (Figure 7). Additionally, the new method leads to a gain of >15% in precision and recall calculated at the family level on a random sample of sequences (Supplementary Table S1). Pfam-N annotated 22.8 million protein sequences that previously had no Pfam annotations, among which >10 million had no annotations from any InterPro member database. Out of >10,000 Swiss-Prot sequences with new Pfam-N matches, >1400 are human proteins.

Pfam-N annotations can be visualized under the ‘Other features’ section of the protein sequence viewer in protein pages on the InterPro website and accessible programmatically through the InterPro API (https://www.ebi.ac.uk/interpro/api/). All the Pfam-N matches can be downloaded through the *extra.xml.gz* file available on the InterPro ftp (https://ftp.ebi.ac.uk/pub/databases/interpro/releases/).

### Discovery of new *S. pombe* orthologs of human and *S. cerevisiae* proteins using Pfam-N

Identifying novel distant orthologs between model species is invaluable, as it can enable the exchange of functional information between proteins that were previously unrecognized as related or provide specific, experimentally testable functional predictions for previously unstudied proteins (28). The manual curation of orthologs between *Schizosaccharomyces pombe* (*S. pombe*) and *Saccharomyces cerevisiae* and between *S. pombe* and humans at the model organism database PomBase focuses on identifying distant orthologs that are missed by automated prediction methods (29). This approach combines automated predictions, biological insights such as co-complex membership, and distant detection methods to create a consensus ortholog dataset with high coverage.

As part of this approach, Pfam-N hits for 94 *Schizosaccharomyces*-specific proteins lacking a known Pfam domain were manually evaluated to uncover additional potential orthologs. Confirmation was based on various criteria: (i) proteins were expected to be conserved; (ii) validation by JackHMMER (1) or Foldseek (30), (iii) similarities in protein features and protein length; (iv) available experimental data, including phenotypes, similar cellular locations, and co-complex membership. This review confirmed the identification of four human proteins and five *S. cerevisiae* proteins as true one-to-one orthologs of *S. pombe* proteins. Among the orthologs identified were the missing peroxisomal proteins *S. cerevisiae* PEX15 (human PEX26), *S. cerevisiae* PEX22, and the missing Mon1-Ccz1 GEF complex subunit (S. *cerevisiae* CCZ1 and human CCZ1). Of the remaining 88 Pfam-N assignments, 16 confirmed previously identified orthologs, 5 confirmed family membership, and 13 provided novel protein family assignments.

Pfam-N also offers the potential to identify further connections between proteins in existing families. For example, the cytochrome c oxidase assembly protein Coa2 (Pfam: PF17051) was determined to be human NDUFA3 (Pfam: PF14987). Since Respiratory Complex I is absent for fission yeast and budding yeast, but assembly factors are conserved, this makes human NDUF3 likely to be an additional assembly factor rather than a core subunit.

## Discussion

The advent of accurate protein structure predictions has been transformational for protein family classification. Having these structural models has been like ‘turning the lights on’, so now we can easily see the extent of individual protein domains and their likely evolutionary relationships. Of course, much of Pfam was created in the dark ages, when experimentally determined structures were few and far between. Thus, we have many legacy entries that require some cleaning up to improve domain boundaries or identify further homologs.

We have seen the power of artificial intelligence (AI) also improving our ability to identify further homologs for families using deep learning methodology. It seems likely that in the coming years, these models will supplant profile-HMMs as the dominant technology for homolog detection. However, at present, the field is developing rapidly, and no single model or technology appears to be an obvious choice to select. Thus, we have already seen three different approaches used to calculate Pfam-N in as many years.

AI, particularly large language models, has the potential to revolutionize the creation of Pfam entries. These technologies can significantly enhance the efficiency and accuracy of generating scientifically current descriptions for entries. For instance, we are currently investigating how AI can streamline the creation of entry descriptions, making the process more time-efficient while ensuring the inclusion of the latest scientific findings. Despite promising initial steps, we are only at the beginning of exploring AI’s capabilities. Substantial further work is required to fully leverage AI’s power in assisting the curation process within Pfam, including refining AI models to better understand and interpret complex biological data and integrating these tools seamlessly into our existing workflows.

Switching off the Pfam website was the first step in a process to reduce the technical debt of the Pfam resource. In the future, our aim is to release Pfam updates more frequently than our current yearly release and to make Pfam simpler and easier to maintain. Our intention is to continue to curate Pfam using the same codebase but to migrate most of our release procedures to become part of the InterPro production cycle. The Pfam updates would then happen at the same time as InterPro releases, every two months. We hope this will benefit the scientific community, as new and updated Pfam entries will be made publicly available more frequently. To achieve this, the underlying version of the UniProtKB RP won’t be updated for every Pfam release; that update process is a time-consuming step of the Pfam release process, and the matches to the whole of UniProtKB will only be calculated during the InterPro release cycle. We hope that these planned changes will enable increased efficiency in Pfam and help it remain a sustainable prospect for the future. Despite all the developments we have seen over the last few years, there remain many protein domain families that are not available in Pfam or any other resource. We hope that the coming years will see significant steps towards a complete and accurate classification of protein families.

## Supplementary Material

gkae997_Supplemental_Files

## Data Availability

Pfam data are accessible through the InterPro website (https://www.ebi.ac.uk/interpro) and downloadable programmatically through the InterPro API (https://www.ebi.ac.uk/interpro/api/entry/pfam) or the Pfam ftp (https://ftp.ebi.ac.uk/pub/databases/Pfam/). The former Pfam website (https://pfam.xfam.org) now hosts a static page that replicates the appearance of the old home page, with its features (search, browse, etc.) redirecting to the corresponding pages on the InterPro website. Pfam-N matches can be downloaded through the extra.xml.gz file available on the InterPro ftp (https://ftp.ebi.ac.uk/pub/databases/interpro/releases/).
