# Correction to ‘The Pfam protein families database: embracing AI/ML’

**DOI:** 10.1093/nar/gkae1276

**Published:** 2024-12-24

This is a correction to: Typhaine Paysan-Lafosse, Antonina Andreeva, Matthias Blum, Sara Rocio Chuguransky, Tiago Grego, Beatriz Lazaro Pinto, Gustavo A Salazar, Maxwell L Bileschi, Felipe Llinares-López, Laetitia Meng-Papaxanthos, Lucy J Colwell, Nick V Grishin, R Dustin Schaeffer, Damiano Clementel, Silvio C E Tosatto, Erik Sonnhammer, Valerie Wood, Alex Bateman, The Pfam protein families database: embracing AI/ML, *Nucleic Acids Research*, 2024; https://doi.org/10.1093/nar/gkae997

In the originally published version of the manuscript there was a typographical error in the last name of the 16th author. This is now emended in the article to read: “Sonnhammer”.

