# Supplementary material for: The Pfam protein families database: embracing AI/ML
Source: Nucleic Acids Res. 2024 Nov 14;53(D1):D523–34. doi: 10.1093/nar/gkae997 (PMC11701544; doi:10.1093/nar/gkae997)
Supplement: gkae997_Supplemental_Files [file gkae997_supplemental_files.zip › supplementary_figure1.pdf]

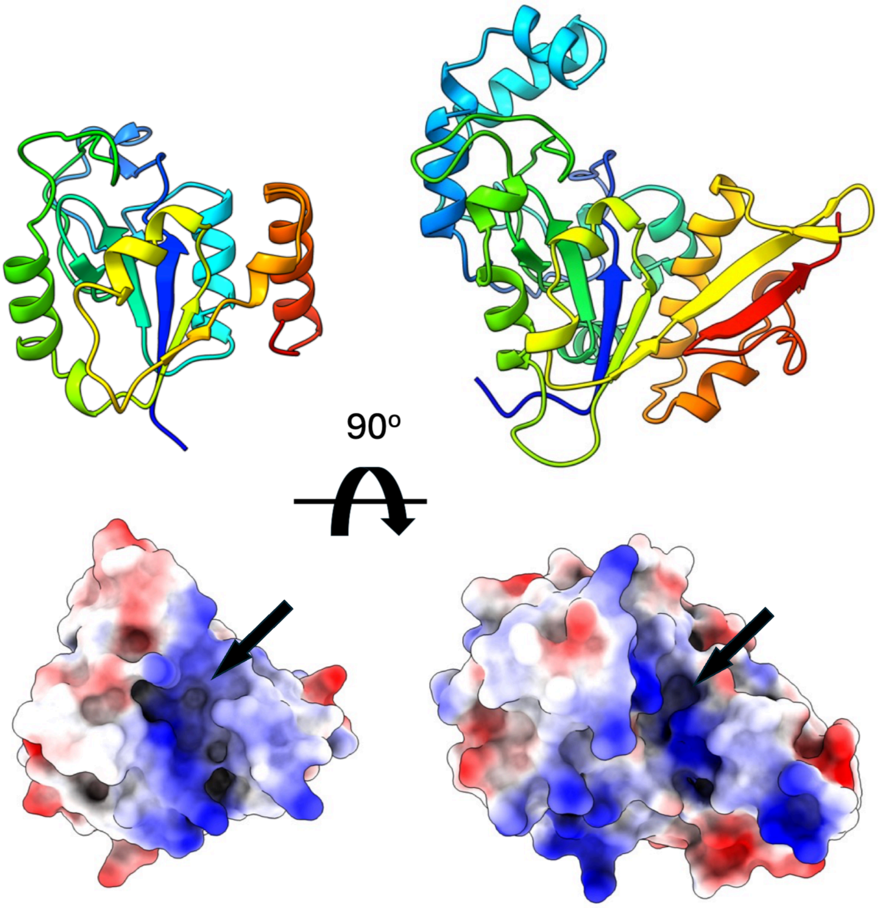

Supplementary Figure 1. Side by side comparison of the AlphaFold2 model of uncharacterized protein YfjM from *Bacillus subtilis* (UniProtKB: O31547), a member of DUF6884 (left) and the crystal structure of DNA-binding protein YaaA from *E. coli* (PDB: 5caj) (right). Top panel: Structures rendered in cartoon and coloured in rainbow (blue to red indicating N- to C-terminal end); Bottom panel: Structures rendered in surface and coloured according to the electrostatic potential (view from the top after ~90° rotation around x-axis). Black arrows indicate the common positively charged cleft, which is likely involved in DNA-binding.
